# Stable organic self-assembled microwire lasers for chemical vapor sensing

**DOI:** 10.1038/s42004-021-00534-x

**Published:** 2021-06-24

**Authors:** Zheming Chen, Chenghu Dai, Wei Xiong, Yanke Che, Chuang Zhang

**Affiliations:** 1 Key Laboratory of Photochemistry, Beijing National Laboratory for Molecular Sciences, Institute of Chemistry, Chinese Academy of Sciences, Beijing, China; 2 University of Chinese Academy of Sciences, Beijing, China

**Keywords:** Physical chemistry, Nanoscale materials

## Abstract

Organic microlasers hold great potentials in fabricating on-chip sensors for integrated photonic circuits due to their chemical versatility and reactivity. However, chemical vapor detection is still challenging for organic microlaser sensors, as it requires not only optical gain and self-assembly capability, but also rapid response to stimuli and long-term stability under high excitation power. In this work, a new laser dye 4,7-bis(9-octyl-7-(4-(octyloxy)phenyl)-9H-carbazol-2-yl)benzo[c][1,2,5]thiadiazole (BPCBT) is designed and synthesized, which self-assembles into microwires showing strong intramolecular charge transfer (ICT) photoluminescence with >80% quantum efficiency. It enables the lasing from BPCBT microwires under a low threshold of 16 μJ·mm^−2^·pulse^−1^ with significantly improved stability over conventional organic microlasers. The stimulated emission amplifies the fluorescence change in the BPCBT microwires under chemical vapors including various acid, acetone, and ethanol vapors, indicating high sensitivity and high selectivity of organic microlaser sensors desirable for compact sensor arrays in integrated photonics.

## Abstract (web summary)

Organic microlaser sensors are highly promising for chemical vapor detection due to their chemical versatility, but their poor stabilities are hampering applications. Here the authors report the synthesis and lasing properties of a dye that is self-assembled into microwires exhibiting a strong acid vapor response and good stability.

## Introduction

Organic micro/nanolasers have drawn great attention because they may serve as functional units such as miniature coherent light source on integrated photonics chips^1–6^. Besides high optical gain coefficient, organic dye molecules take advantages of chemical versatility as well as sensitivity to external stimuli compared with their inorganic counterpart^7–9^, which extends their potential applications from microlasers to on-chip chemical sensors. The arrays of organic microlasers could be prepared by various solution-processable methods^4,10,11^, providing opportunities for efficient and sensitive identification of chemicals in complicated environments. In principle, the sensitivity of these microlaser sensors would be significantly enhanced compared with that of traditional fluorescent sensors according to the light amplification nature of the stimulated emission process^12–14^. However, high-intensity excitation is usually required for laser action^15^, and thus the stability would become an essential issue in the fabrication of chemical sensors based on organic microlasers^16,17^. In addition, the excited state of these chromophores should be both highly efficient in radiative decay process and highly reactive to the introduction of trace analyte^18,19^. Rational design on molecular structures that combines the capabilities of lasing and sensing in excited-state processes is therefore crucial for the realization of chemical vapor detection based on organic microlasers.

Intramolecular charge transfer (ICT), in which the electron transfers from the donor to the acceptor moieties in the same molecule, can prolong the photoexcitation lifetime towards the construction of various multi-functional luminescent molecules^20,21^. Self-assembled microstructures of ICT molecules have been demonstrated to serve as low-threshold microlasers that may switch the output wavelength upon temperature or pump energy changes^22,23^. It has also been proved that the ICT complex may show superior performance towards continuous-wave pumping lasers by partially overcoming the optical loss of triplet accumulation^24^. The building blocks of donor and acceptor provide great possibilities in involving functional groups for chemical sensing in the molecular structures while maintaining the high optical gain for laser action^25,26^. More interestingly, the competition between radiative and nonradiative excited-state processes in ICT molecules would lead to a leverage effect on the population distribution of photoexcitations and thus the enhancement of sensitivity via stimulated emission^26^. This design strategy of ICT molecules offers a promising approach to microlaser-based chemical vapor sensors, as well as other functional on-chip units using organic microlasers in chemo/bioanalytical and environmental fields^27^.

**Fig. 1:**
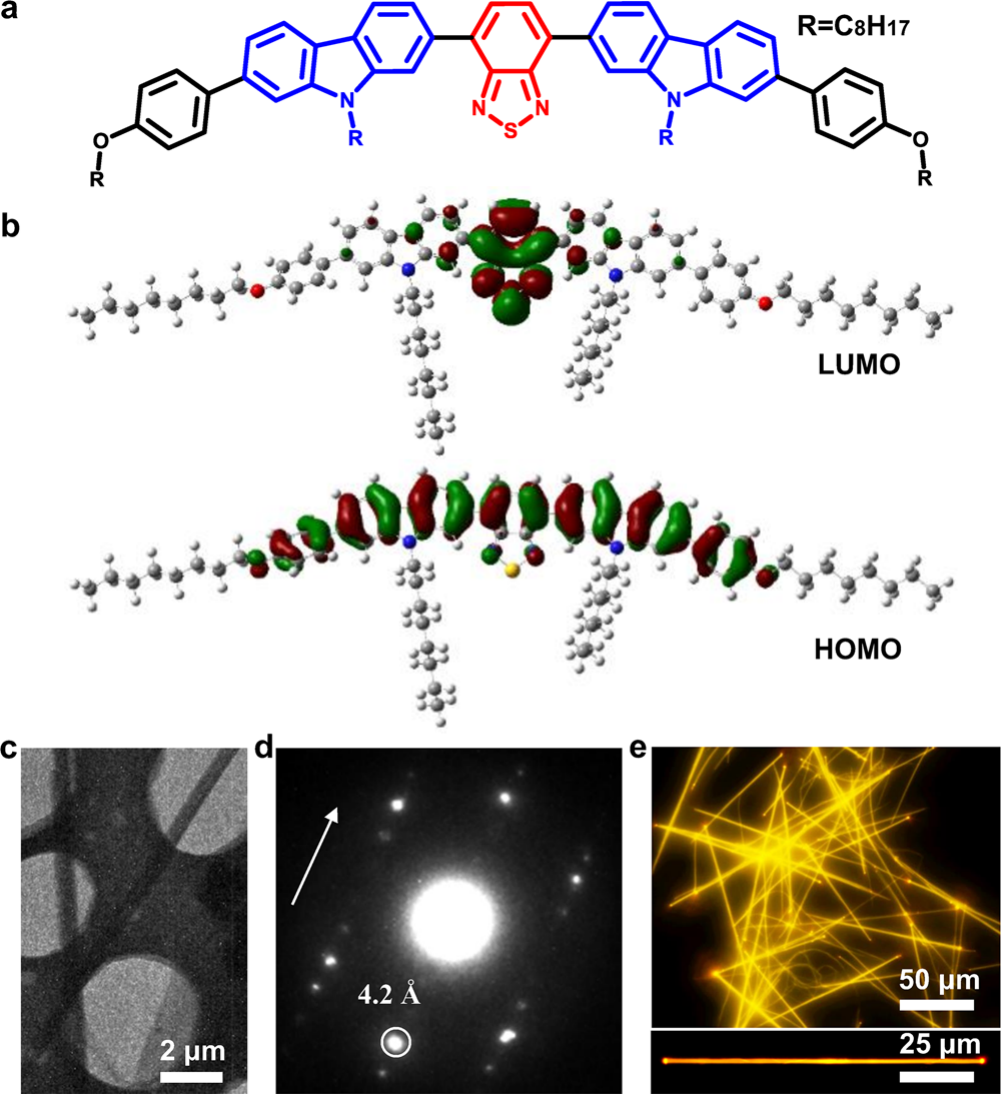
Molecular design and characterization of BPCBT microwires. **a** Chemical structure and **b** molecular orbital diagrams (LUMO and HOMO) of BPCBT. **c** TEM image of BPCBT microwires. **d** Corresponding SAED pattern. **e** Fluorescence microscopy images of large-area BPCBT microwires and a single microwire.

**Fig. 2:**
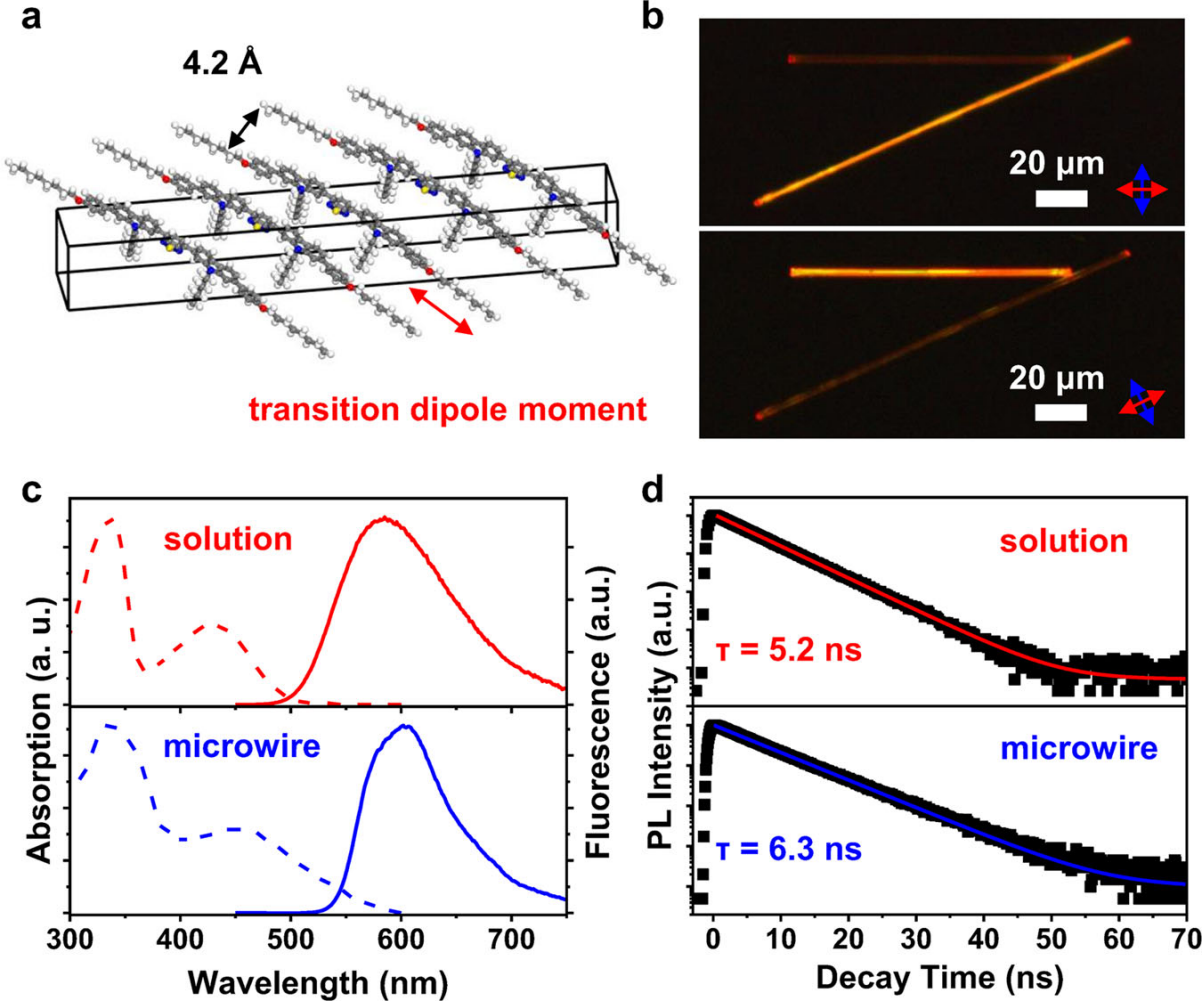
Packing style and fluorescence properties of BPCBT microwires. **a** Schematic illustration of molecular packing and TDM direction in the BPCBT microwire. **b** POM images of two crossed microwires. The arrows indicate the direction of optical polarizers. **c** Absorption and photoluminescence spectra of the dilute solution (red) and the microwire (blue) of BPCBT. Excitation wavelength in photoluminescence measurement is 400 nm. **d** Photoluminescence decay curves from the BPCBT solution and the microwires. Excitation and emission wavelengths are 400 and 585 nm, respectively.

In this study, we have designed and synthesized a new ICT molecule 4,7-bis(9-octyl-7-(4-(octyloxy)phenyl)-9H-carbazol-2-yl)benzo[c][1,2,5]thiadiazole (BPCBT) (see Supplementary Methods, Supplementary Scheme 1, Figs. 1 and 2) with a nearly 100% photoluminescence quantum yield (PLQY). The ICT occurs from the carbazole derivative donor to the benzothiadiazole acceptor, and has been investigated by density functional theory (DFT) calculations and steady-state spectroscopic experiments. The side alkyl chains enlarge the intermolecular distance in the self-assembled microwire structures of BPCBT molecules and avoid the possible aggregation-induced quenching. The BPCBT microwires that show over 80% PLQY can work as microlasers with a low threshold 16 μJ·mm^−2^·pulse^−1^ and superior stability beyond conventional dye-doped microlasers. As the carbazole group may form nonradiative charge-transfer state with external proton donors, the single microwire is chemically sensitive to acid vapors, and the change in its lasing intensity is dramatically enhanced compared with that in spontaneous fluorescence. Moreover, the wettability of solvent vapors leads to the morphological change of BPCBT microwires as optical microcavities, which extends the species of organic vapors for these microlaser sensors to non-reactive compounds such as acetone and ethanol.

## Results and discussion

### Preparation of BPCBT microwires

In the BPCBT molecule, the benzothiadiazole unit is sandwiched between two carbazole groups resulting in a symmetrical D-A-D structure (Fig. 1a). The rigid carbazole group is used for stable UV light absorber that has been used in blue light-emitting diodes^28^, as well as in active moiety to iodide and explosive detection^29,30^. While the benzothiadiazole serves as a high-efficient fluorescent core with electron-withdrawing capacity that has applications in bioimaging and electroluminescence systems^31,32^. Thereby, the charge transfer between carbazole donor and benzothiadiazole acceptor allows for the formation of highly emissive excited states with remarkable photostability. In addition, the long alkyl groups improve the solubility of BPCBT molecules as well as protect the delocalized charge-transfer states from being quenched by strong electronic coupling with enhancing intermolecular distance^33,34^.

DFT calculations were performed to study the geometric and electronic structures of BPCBT molecule. Frontier orbital analyses (Fig. 1b) show that the highest occupied molecular orbital (HOMO) is mainly located on molecular skeleton, while the lowest unoccupied molecular orbital (LUMO) is located on electron-withdrawing benzothiadiazole unit. The charge-transfer feature of BPCBT molecule reduces the energy gap to ~2.70 eV compared with those of benzothiadiazole (~4.24 eV) and carbazole (~4.79 eV) molecules (Supplementary Fig. 3). As the solvent effects and the intermolecular interactions are not involved in the calculation^35^, these LUMO and HOMO energies obtained are not the same as the experimental values obtained from cyclic voltametric measurement (Supplementary Fig. 4). The time-dependent DFT calculation shows that the transition dipole moment (TDM) is almost parallel to the long axis of BPCBT molecule (Supplementary Fig. 5), and the benzothiadiazole and carbazole units present a distorted conformation with a dihedral angle of ~33° while the dihedral angle of carbazole and benzene rings is ~37° (Supplementary Fig. 6). As a result, the overlapping of intermolecular π-conjugated electrons is obviously reduced, and the dipole–dipole interaction between neighboring BPCBT molecules becomes dominated and facilitates the molecular packing in a preferential direction^36^.

The BPCBT microwires were prepared by an anti-solvent vapor diffusion method (see details in the “Methods” section)^37^. As shown in Fig. 1c, transmission electron microscopy (TEM) image indicates that these self-assembled microwires are highly crystalline and the corresponding selected area electron diffraction (SAED, Fig. 1d) pattern proves face-to-face distance is 4.2 Å with small angle along the direction of microwire. This agrees with the signal that appears at ~22.1° in the X-ray diffraction (XRD) measurements (Supplementary Fig. 7), which is attributed to the preferential molecular packing based on dipole–dipole interaction. The other two structural parameters (17.3 Å, 39.1 Å) are different from the size of BPCBT molecule (14.9 Å, 51.4 Å), indicating that the molecules incline in the microwire with the side chains standing on the substrate. Figure 1e presents the fluorescence microscopy image of self-assembled microwires showing bright yellow emission. A representative single microwire exhibits optical waveguiding property as the ends are much brighter than the body of microwire^38^.

### Photoluminescence and lasing properties

The packing style of BPCBT molecules in the microwire is illustrated in Fig. 2a, in which the molecular backbones are aligned in parallel and the angle with respect to the long axis of microwire is ~20°. This unidirectional molecular alignment was demonstrated by the birefringence analysis of microwires under polarized optical microscope (POM), as shown in Fig. 2b. The two crossed microwires turn bright and dark alternately upon the rotation of polarizers^39^, and the high contrast shows that the defect density in these single-crystalline microwires is extremely low. Note that the maximum intensity in the polarized photoluminescence profile appeared at a cross angle of ~20° (Supplementary Fig. 8), which agrees with the direction of TDM in the microwire. The above features are beneficial to the properties of high-efficiency photoluminescence and optical waveguiding with a relatively low propagation loss (Supplementary Fig. 9) in BPCBT microwires. Besides, the light signal from a single BPCBT microwire could be efficiently coupled into neighboring wires (Supplementary Fig. 10).

As shown in Fig. 2c, the dichloromethane solution of BPCBT (10^−5^ M) presented two absorption bands located at ~340 and ~430 nm, corresponding to the ^1^L_b_ ← ^1^A transition of carbazole group^40^ and the ICT transition of BPCBT, respectively. In comparison, the PL spectrum shows only the feature of ICT transition which is centered at ~585 nm with a large Stokes shift^41,42^. The absorption of BPCBT misscrowires is similar to that of its dilute solution expect for a tail toward longer wavelength due to light scattering^43^. The photoluminescence band of microwires becomes narrower compared with that of BPCBT solution, because the confinement of geometric configurations reduces the degree of freedom of molecular vibrations^44^. No intermolecular transition is observed and the ICT transition is still predominated in microwires, and the PLQY of BPCBT microwires is over 80% (Supplementary Fig. 11) which is close to the near-unity PLQY of monomers. It attributes to steric hindrance effects enlarging intermolecular distances, keeping intrinsic monomer emission. As a result, the photoluminescence lifetime (Fig. 2d) of BPCBT microwires (~6.3 ns) is also close to that of solution (~5.2 ns), as the π-electron coupling between neighboring molecules is quite weak in the microwire.

**Fig. 3:**
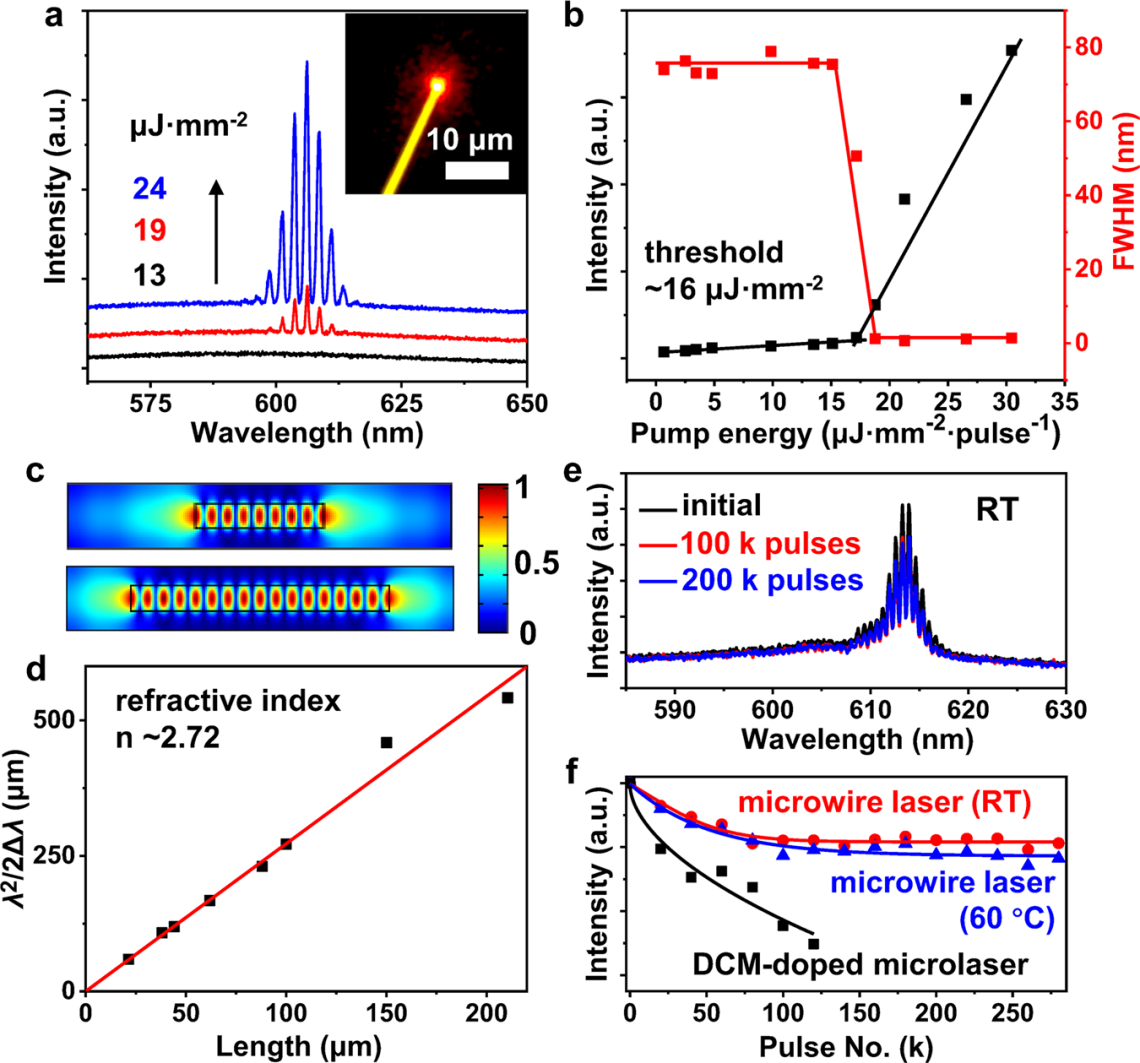
Lasing properties of BPCBT microwires. **a** Emission spectra from a single BPCBT microwire under different pump fluences of a 400 nm pulsed laser (~200 fs, 1 kHz). Inset is optical microscopy image of microwire tip. **b** Emission intensity (black) and FWHM (red) as a function of pump energy. **c** Electric-field distributions in the microwires (top, 1-μm length; bottom, 2-μm length) as FP-type optical resonators. **d** Plots of *λ*^2^/2Δ*λ* versus the length of microwires and the linear fit according to FP-type resonance. **e** Lasing spectra of microwire at initial state, after 100 k pulses and 200 k pulses at RT. **f** Lasing intensities of microwire at RT and 60 °C during a series of excitation laser pulses. The DCM-doped microsphere laser was used as a control sample.

The high PLQY and relatively long excited-state lifetime of BPCBT microwires are important to achieve laser action at low threshold, and the lasing characterization of single microwires was performed on a home-built optical microscope system equipped with a pulsed laser source (Supplementary Fig. 12). As shown in Fig. 3a, a broad spontaneous emission band was observed from the wire tip at a low pump fluence (~13 μJ·mm^−2^·pulse^−1^). A series of sharp peaks emerged on top of the broad band when the excitation energy was increased to ~19 μJ·mm^−2^·pulse^−1^. Diffraction pattern appeared around the wire tip under a high pump fluence (~24 μJ·mm^−2^·pulse^−1^) which indicates a typical Fabry-Pérot (FP) type resonance along the microwire^45^. The microwire laser shows a threshold at ~16 μJ·mm^−2^·pulse^−1^ in the excitation power dependence profile, accompanied with a sharp decrease from 75 to 1 nm in the full width at half maximum (FWHM) of emission band (Fig. 3b). The performance of microlasers based on BPCBT microwires is highly comparable to previously reported laser dyes of carbozole derivatives (Supplementary Table 1). The electric-field distributions of FP-type microcavities (Fig. 3c) indicate that the resonant modes can be described as Δ*λ* = *λ*^2^/2*nL*, where Δ*λ*, *λ*, *n*, and *L* are mode spacing, wavelength, refractive index, and length of microwire, respectively^46^. Accordingly, the mode spacing Δ*λ* decreases with the increase in the length of microwire *L* (Supplementary Fig. 13), and the refractive index calculated from the theoretical fitting with FP-type optical resonance is ~2.72 (Fig. 3d), which is higher than that of normal organic crystals due to the close molecular packing along the microwire axis^47^.

The ultralow threshold is essential for the improved operation stability of BPCBT microlasers. As shown in Fig. 3e, the lasing spectra decreased by ~20% after the first 100 k pulses but remained almost unchanged during the excitation of 100 to 200 k pulses, which indicates that the microwire laser can keep stable multi-mode lasing output during the operation. Figure 3f shows the time-dependent output intensities from BPCBT microlasers, in which the operations at room temperature (RT) and 60 °C would not cause severe degradation of BPCBT chromophores in the microwires. A polymeric microlaser, [4-(dicyanomethylene)-2-methyl-6-(4-dimethylaminostyryl)-4H-pyran] (DCM) doped polystyrene microsphere, was prepared as a control sample to demonstrate the superior stability of BPCBT microlasers. The rigid moiety and nonplanar structure of BPCBT molecule facilitate its thermal and photochemical stability which is essential to the reliable sensing of chemical vapors.

### Chemical vapor detection

**Fig. 4:**
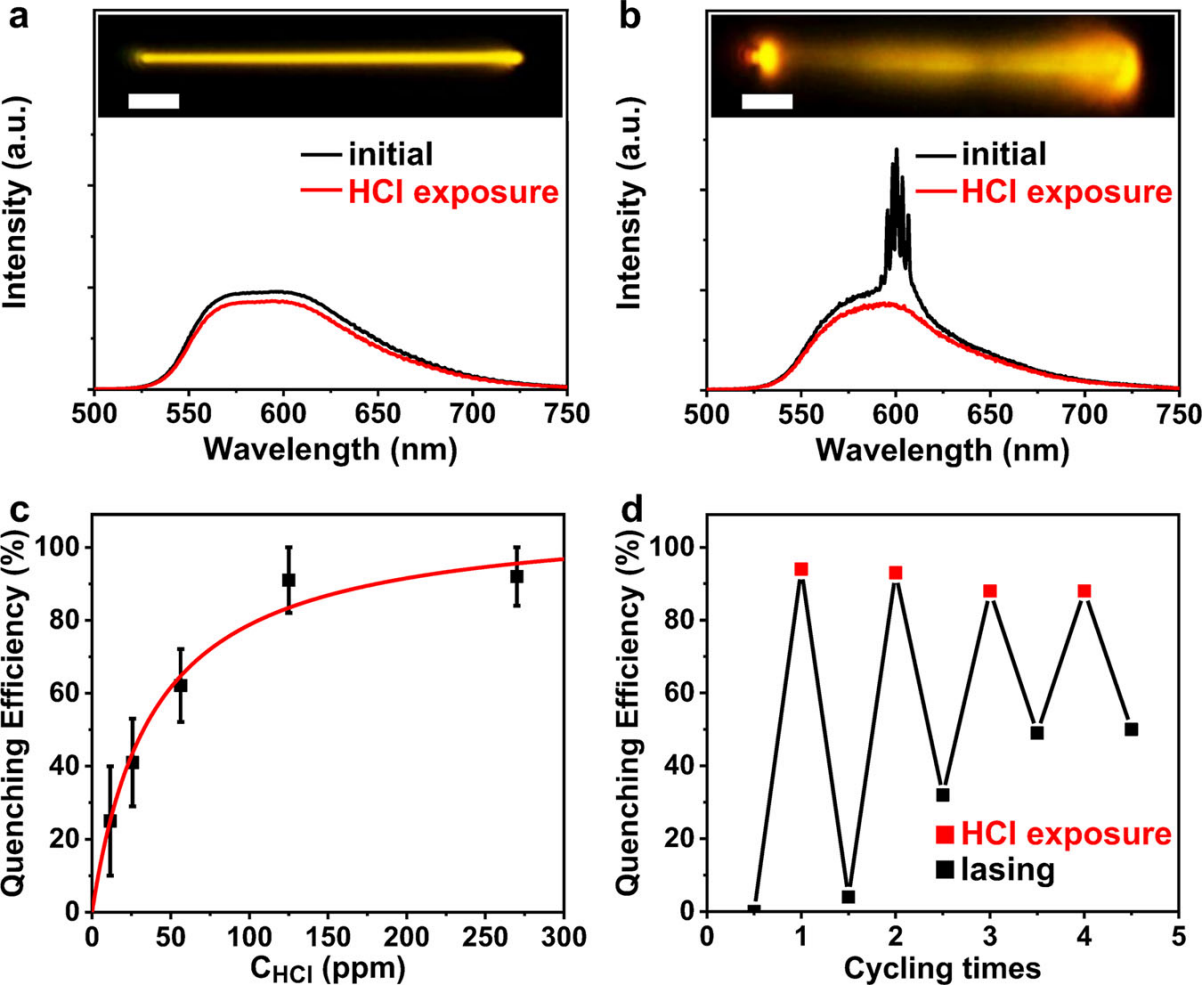
Sensing properties of BPCBT microwires to HCl vapor. **a** Photoluminescence and **b** lasing spectra of BPCBT microwires before and after exposure to HCl vapor. Insets are optical microscopy images of microwires. Scale bars are 5 μm. **c** Quenching efficiency of lasing from a BPCBT microwire upon exposure to HCl vapor with different concentrations. Error bars represent the deviation of three measurements. **d** Cycles of lasing quenching and recovery from a BPCBT microwire upon exposure to HCl vapor and air with 10 min intervals at constant pump energy.

The carbazole group is known to be sensitive to protonic acids via the protonation interaction^48^. This allows for the detection of acid vapor by monitoring the change in emission intensity from BPCBT microwires. By adding several drops of HCl into BPCBT solution, an immediate decrease in fluorescence intensity was detected as nonradiative decay was introduced by the interaction between BPCBT and HCl (Supplementary Fig. 14). However, the response of single BPCBT microwire when being exposed to saturated HCl vapor becomes quite limited (<10%), as shown in Fig. 4a. This is probably because the HCl cannot penetrate into the body and only can be adsorbed to the surface which would not be able to completely quench the photoluminescence of microwire (Supplementary Fig. 15). Interestingly, the lasing emission from BPCBT microwire excited above threshold totally disappeared upon the exposure to HCl vapor (Fig. 4b), which demonstrates the enhancement of sensitivity due to the amplification through stimulated emission process. The limiting concentration of HCl to completely quench lasing is as low as ~100 ppm (Fig. 4c). In addition, the lasing can recover in a short time and remains sensitive to HCl vapor repeatedly (Fig. 4d).

**Fig. 5:**
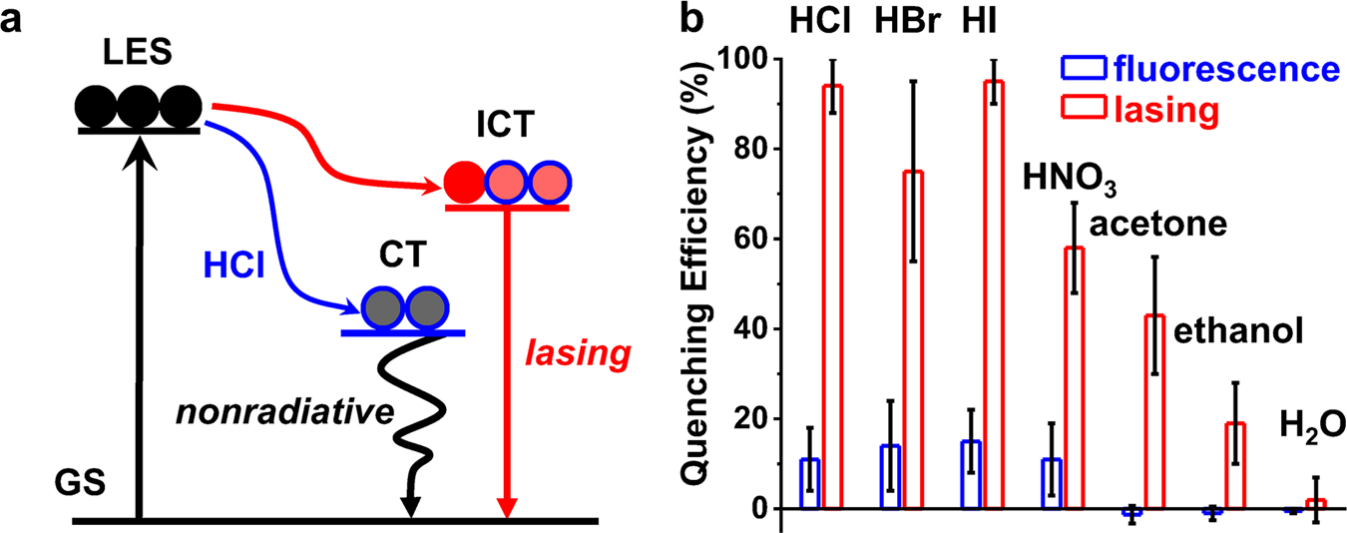
Enhanced sensing properties of BPCBT microwires to chemical vapors. **a** Mechanism of lasing sensor based on the competition between nonradiative transition of charge transfer (CT) to HCl and radiative ICT transition of BPCBT. **b** Quenching efficiencies of photoluminescence and lasing from BPCBT microwires upon exposure to chemical vapors of HCl, HBr, HI, HNO_3_, acetone, ethanol, and water, respectively. Error bars represent the deviation of three measurements.

As illustrated in Fig. 5a, the absorption of incident ultraviolet photons would excite the ground state (GS) to the local excited state (LES) of carbazole groups, and consequently generate the ICT state between carbazole and benzothiadiazole in BPCBT molecules. The laser action of BPCBT microwires may take place when the population inversion condition is fulfilled. When HCl is adsorbed to the microwire, a newly formed excited state upon the charge transfer (CT) from carbazole to HCl provides an alternative nonradiative decay route to the LES of carbazole groups. In this case, the population inversion is no longer fulfilled and thus the laser action from BPCBT microwire is turned off. As shown in Fig. 5b, the quenching efficiency of lasing intensity upon HCl exposure is increased by ~10 times compared with that of photoluminescence from BPCBT microwires. The significant enhancements on sensing properties of BPCBT microwires to various acid vapors (e.g., HBr, HI, and HNO_3_) were also demonstrated (Supplementary Figs. 16–18). In comparison, the vapors of organic solvents (acetone and ethanol) cannot quench the photoluminescence; however, a change in lasing output was detected upon the adsorption of solvent molecules on the microwire. The lasing modes also varied after the exposure to solvents (Supplementary Fig. 19) as the FP-type optical resonator of microwires is influenced. This interpretation was further verified by the variation of lasing modes upon the temperature change (Supplementary Fig. 20). The sensitivity to acetone vapor is slightly higher than that to ethanol, because the BPCBT microwires can be dissolved by acetone but not ethanol. Note that both photoluminescence and lasing are immune to exposure in H_2_O vapor as the surface of BPCBT microwires is hydrophobic. Therefore, the lasing process in BPCBT microwires not only enhances the sensitivity but also improves the selectivity to chemical vapors, showing the potentials of organic microlasers in the fabrication of on-chip multi-channel sensor arrays for complex analytes^49^.

### Conclusion

In summary, the luminescent ICT molecule named BPCBT was designed and synthesized with high optical gain, high stability, and chemical sensitivity. The self-assembled microwires of BPCBT molecules take advantages of high PLQY and large Stokes shift, and can work as organic microlasers with a low threshold of ~16 μJ·mm^−2^·pulse^−1^. The superior stability of laser action from BPCBT microwires enables the detection of various chemical vapors including various acid vapors, acetone, and ethanol with these microlasers. The enhanced sensitivity and improved selectivity of lasing sensors based on BPCBT microwires opens up a new avenue to the on-chip chemical detectors in integrated photonics applications.

## Methods

### Preparation of BPCBT microwires

BPCBT microwires were prepared by a one-step anti-solvent vapor diffusion method^37^. Typically, the BPCBT compound (1 mg) was dissolved into a mixture of dichloromethane and methanol (1:1, 50 mL), and a vial of transparent solution (2 × 10^−5^ M) was obtained after stirring for 15 min. Then a vial of 1 mL obtained solution was placed in a closed beaker containing ethanol (3 mL) as the anti-solvent. After 3 h the suspension of BPCBT microwires was obtained in the vial. The size of microwires increased with the increase of aging time. The suspension was drop-casted on the substrates for further characterizations. In addition, the DCM-doped polystyrene microspheres were prepared by an emulsion-solvent-evaporation method according to previous report^50^.

### Measurements

The analysis of structure and crystallinity of BPCBT microwires was carried out on a transmission electron microscope (JEOL JEM-1011) and an X-ray diffractometer (Panalytical Empyrean) with Cu Kα radiation (*λ* = 1.54056 Å). The cyclic voltammetric measurement was conducted with Pt disk and Pt wire as the working and counter electrodes, Ag/AgCl electrode (sat. KCl) as the reference electrode, and *n*-Bu_4_NPF_6_ (0.1 M) as supporting electrolyte. The fluorescence images were taken on an inverted optical microscope (Nikon Ti-U) excited with the UV band (330–380 nm) of a mercury lamp. The polarized dark-field optical microscopy images were captured with an upright polarizing microscope (Nikon ECLIPSE Ci-POL). The absorption and photoluminescence spectra were obtained from a UV-visible-NIR spectrophotometer (Hitachi UH4150) and a fluorescence spectrophotometer (Hitachi, F-7000), respectively. The absolute quantum yields of BPCBT solution and microwires were recorded on an absolute quantum yield spectrometer (Hamamatsu C11347) equipped with an integrating sphere apparatus and a 150 W CW Xenon light source. The decay curves of photoluminescence were measured with a Quantaurus-Tau fluorescence lifetime spectrometer (Hamamatsu Photonics C11367-31). Optically pumped lasing measurements were carried out on a home-built far-field micro-PL system. For sensing of chemical vapors, the BPCBT microwires and chemical vapors were sealed in a 3D printed chamber to provide an atmosphere with constant vapor concentration. Before each sensing measurement, the air flow was injected to evacuate residual vapors in the chamber. The spectra were collected through an inverted optical microscope before/after the exposure of chemical vapors, and further analyzed to evaluate the sensing properties.

### Calculations

The molecular geometry was optimized by Gaussian 09^51^ at B3lyp/6-31G** level in the gas phase, and the molecular orbitals were calculated using time-dependent density functional theory (TDDFT) at B3lyp/6-31G** level. A commercial finite-element-methods-based software (Comsol Multiphysics 5.0) was used for the numerical simulations. The sizes of calculation regions were 1 × 0.2 µm^2^ and 2 × 0.2 µm^2^ using a scattering boundary condition with a background refractive index of air (*n* = 1.0), and the refractive indexes of the wires were set to 2.72 at 606 nm.

### Reporting summary

Further information on research design is available in the Nature Research Reporting Summary linked to this article.

## Supplementary information

Supplementary Information

Reporting Summary

## Data Availability

The main data supporting the finding of this study are available within the paper and its Supplementary Information file. Other relevant data are available from the corresponding author upon reasonable request.

## Code availability

The Gaussian 09 package, Revision B.01 was employed for all the DFT calculations. The software and instructions for its use are available at https://gaussian.com/. The Comsol Multiphysics 5.0 was used for numerical simulations. The software and instructions for its use are available at https://cn.comsol.com/.
