# Supplementary material for: Stable organic self-assembled microwire lasers for chemical vapor sensing
Source: Commun Chem. 2021 Jun 24;4:97. doi: 10.1038/s42004-021-00534-x (PMC9814925; doi:10.1038/s42004-021-00534-x)
Supplement: Supplementary file 1 — Supplementary Information [file 42004_2021_534_MOESM1_ESM.pdf]

Supplementary Information for

Stable organic self-assembled microwire lasers for chemical vapor sensing

Zheming Chen[1,2,†], Chenghu Dai[1,†], Wei Xiong[1], Yanke Che[1,*] and Chuang Zhang[1,*]

[1]Key Laboratory of Photochemistry, Beijing National Laboratory for Molecular Sciences, Institute of Chemistry, Chinese Academy of Sciences, Beijing 100190, China

[2]University of Chinese Academy of Sciences, Beijing 100049, China

[†]These authors contributed equally to this work.

*E-mail: zhangc@iccas.ac.cn; ykche@iccas.ac.cn

Contents

Supplementary Methods.

Supplementary Scheme 1. Synthesis route of BPCBT.

Supplementary Figure 1. ^{1}H NMR spectrum of BPCBT.

Supplementary Figure 2. MALDI-MS of BPCBT.

Supplementary Figure 3. Molecular orbitals and energy level diagrams of benzothiadiazole, BPCBT and carbazole.

Supplementary Figure 4. Cyclic voltammograms of BPCBT in solution.

Supplementary Figure 5. Transition dipole moment of BPCBT.

Supplementary Figure 6. Dihedral angles and molecular lengths of BPCBT.

Supplementary Figure 7. XRD pattern of BPCBT microwires.

Supplementary Figure 8. Polarized fluorescence intensity of the BPCBT microwire.

Supplementary Figure 9. Optical waveguiding property of BPCBT microwire.

Supplementary Figure 10. Optical waveguiding property in coupled BPCBT microwires.

Supplementary Figure 11. Absolute fluorescence quantum yields of BPCBT monomers and microwires.

Supplementary Figure 12. Schematic illustration of the home-built setup for optical characterization.

Supplementary Figure 13. Lasing spectra above threshold from BPCBT microwires with various lengths.

Supplementary Figure 14. Photoluminescence spectra from BPCBT solution before and after adding HCl.

Supplementary Figure 15. Photoluminescence spectra from BPCBT film before and after exposure to HCl vapor.

Supplementary Figure 16. Photoluminescence and lasing spectra from BPCBT microwires before and after exposure to HBr vapor.

Supplementary Figure 17. Photoluminescence and lasing spectra from BPCBT microwires before and after exposure to HI vapor.

Supplementary Figure 18. Photoluminescence and lasing spectra from BPCBT microwires before and after exposure to HNO_3 vapor.

Supplementary Figure 19. Lasing spectra of a BPCBT microwire before and after exposure to acetone vapor.

Supplementary Figure 20. Lasing spectra from a BPCBT microwire at different temperatures.

Supplementary Table 1. Laser actions in carbozole derivatives and BPCBT.

Supplementary Methods

Synthesis of BPCBT molecule

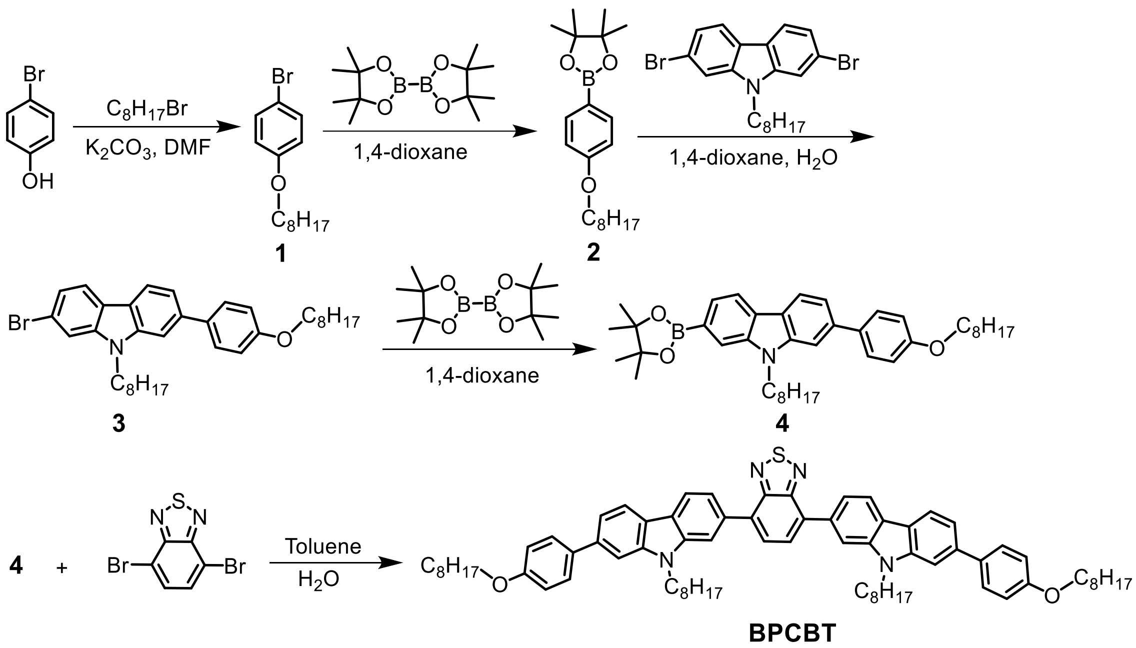

Supplementary Scheme 1. Synthesis route of BPCBT.

*Synthesis of 1-bromo-4-(octyloxy)benzene (**1**)*: 4-bromophenol (2.0 g, 11.56 mmol), 1-bromooctane (2.68 g, 13.87 mmol) and potassium carbonate (4.78 g, 34.68 mmol) were added into deoxygenated N,N-Dimethylformamide (40 mL). Then the mixture was heated to 120 °C and stirred overnight under Ar. After cooling down to the room temperature, 100 mL water was poured into the above solution and the mixture was extracted with ethyl acetate (3 × 60 mL). The combined organic layer was washed with brine (saturated) for three times, dried over Na_2SO_4, and concentrated under vacuum. The residue was purified by column chromatography on the silica gel (petroleum as the eluent) to afford **1** (3.1 g, 10.9 mmol, 94% yield).

Molecule **1**: ^{1}H NMR (400 MHz, $CDCl_3$): δ 7.34 (d, *J* = 8 Hz, 2 H), 6.76 (d, *J* = 8 Hz, 2 H), 3.91 (t, *J* = 8 Hz, 2 H), 1.73-1.80 (m, 2 H), 1.4-1.28 (m, 10 H), 0.88 (t, *J* = 8 Hz, 3 H).

*Synthesis of 4,4,5,5-tetramethyl-2-(4-(octyloxy)phenyl)-1,3,2-dioxaborolane (**2**)*: A mixture of **1** (3.1 g, 10.9 mmol), potassium acetate (5.3 g, 54.5 mmol), bis(pinacolato)diboron (3 g, 12 mmol), and Pd(dppf)Cl_2 (415 mg, 0.545 mmol) in deoxygenated 1,4-dioxane (60 mL) was heated to 80 °C and stirred overnight under Ar. The solvent was evapourated under vacuum. The residue was poured into water (80 mL) and extracted with ethyl acetate (3 × 40 mL). The combined organic layer was washed with brine (saturated), dried over Na_2SO_4, and concentrated under vacuum. The residue was purified by column chromatography on the silica gel (petroleum:ethyl acetate = 20:1 as the eluent) to afford **2** (3.3 g, 10 mmol, 91% yield).

Molecule **2**: ^{1}H NMR (400 MHz, $CDCl_3$): δ 7.74 (d, *J* = 8 Hz, 2 H), 6.89 (d, *J* = 8 Hz, 2 H), 3.97 (t, *J* = 8 Hz, 2 H), 1.82-1.73 (m, 2 H), 1.45-1.28 (m, 22 H), 0.88 (t, *J* = 8 Hz, 3 H).

*Synthesis of 2-bromo-9-octyl-7-(4-(octyloxy)phenyl)-9H-carbazole (**3**)*: Aqueous solution of potassium carbonate (2 M, 4 mL) was added to a stirred solution of **2** (760 mg, 2.29 mmol), 2,7-dibromo-9-octyl-9H-carbazole (1 g, 2.29 mmol), and tetrakis(triphenylphosphine) palladium (131 mg, 0.11 mmol) in deoxygenated mixed solution of toluene (20 mL). Then the mixture was heated to 80 °C overnight under Ar. After removal of the solvent under vacuum, the residue was extracted with dichloromethane (3 × 30 mL) and water (40 mL). The combined organic layers were washed with brine (saturated), dried over Na_2SO_4, and concentrated under vacuum. The residue was purified by column chromatography (silica, petroleum:dichloromethane = 10:1) to afford **3** (550 mg, 43% yield).

Molecule **3**: ^{1}H NMR (400 MHz, $CDCl_3$): δ 8.07 (d, *J* = 8 Hz, 1 H), 7.93 (d, *J* = 8 Hz, 1 H), 7.64-7.60 (m, 2 H), 7.54-7.50 (m, 2 H), 7.45 (dd, J_1 = 8 Hz J_2 = 1.6 Hz, 1 H), 7.34 (dd, J_1 = 8 Hz J_2 = 1.6 Hz, 1 H), 7.03 (d, *J* = 8 Hz, 2 H), 4.28 (t, *J* = 8 Hz, 2 H), 4.02 (t, *J* = 8 Hz, 2 H), 1.90-1.80 (m, 4 H), 1.79-1.22 (m, 20 H), 0.94-0.84 (m, 6 H).

*Synthesis of 9-octyl-2-(4-(octyloxy)phenyl)-7-(4,4,5,5-tetramethyl-1,3,2-dioxaborolan-2-yl)-9H-carbazole (**4**)*: A mixture of **3** (510 mg, 0.91 mmol), potassium acetate (446 mg, 4.56 mmol), bis(pinacolato)diboron (278 mg, 1.1 mmol), and Pd(dppf)Cl_2 (35 mg, 0.046 mmol) in deoxygenated 1,4-dioxane (20 mL) was stirred overnight at 80 °C under Ar. After removal of the solvent under vacuum, the residue was extracted with dichloromethane (3 × 30 mL) and water (30 mL). The combined organic layers were washed with saturated NaCl solution, dried over Na_2SO_4, and concentrated under vacuum. The residue was purified by column chromatography (silica, petroleum:dichloromethane = 2:1) to afford **4** (470 mg, 85% yield).

Molecule **4**: ^{1}H NMR (400 MHz, $CDCl_3$): δ 8.11 (dd, J_1 = 12 Hz J_2 = 8 Hz, 2 H), 7.87 (s, 1 H), 7.70 (d, *J* = 8 Hz, 1 H), 7.65 (d, *J* = 8 Hz, 2 H), 7.52 (s, 1 H), 7.43 (dd, J_1 = 8 Hz J_2 = 1.6 Hz, 1 H), 7.03 (d, *J* = 8 Hz, 2 H), 4.37 (d, *J* = 8 Hz, 2 H), 4.03 (d, *J* = 8 Hz, 2 H), 1.92-1.79 (m, 4 H), 1.51-1.25 (m, 20 H),1.47 (s, 12 H), 0.92-0.87 (m, 6 H).

*Synthesis of 4,7-bis(9-octyl-7-(4-(octyloxy)phenyl)-9H-carbazol-2-yl)benzo[c][1,2,5] thiadiazole (**BPCBT**)*: To a stirred solution of **4** (430 mg, 0.7 mmol), 4,7-dibromobenzo[c][1,2,5]thiadiazole (97 mg, 0.33 mmol), tetrabutylammonium bromide (10.6 mg, 0.033 mmol), and tetrakis(triphenylphosphine)palladium (20 mg, 0.016 mmol) in deoxygenated toluene (30 mL), aqueous solution of potassium carbonate (5 mL, 2.0 M) was added. Then the mixture was heated to 95 °C and stirred overnight under Ar. The solvent was evaporated under vacuum. The residue was poured into water (50 mL) and extracted with dichloromethane (3 × 50 mL). The combined organic layer was washed with brine (saturated), dried over Na_2SO_4, and concentrated under vacuum. The residue was purified by column chromatography on the silica gel (petroleum:dichloromethane = 1:1 as the eluent) to afford **BPCBT** (260 mg, 70% yield). The resulting target compound was confirmed by ^{1}H NMR and MALDI-MS as below.

Molecule **BPCBT**: ^{1}H NMR (400 MHz, $CDCl_3$): δ 8.24 (d, J = 8 Hz, 2 H), 8.17 (d, J = 8 Hz, 2 H), 8.12 (s, 2 H), 7.99 (s, 2 H), 7.83 (d, J = 8 Hz, 2 H), 7.67 (d, J = 8 Hz, 4 H), 7.57 (s, 2 H), 7.46 (d, J = 8 Hz, 2 H), 7.03 (d, J = 8 Hz, 4 H), 4.44 (t, J = 8 Hz, 4 H), 4.04 (t, J = 8 Hz, 4 H), 1.99-1.94 (m, 4 H), 1.87-1.80 (m, 4 H) 1.50-1.24 (m, 40 H), 0.91-0.82 (m, 12 H). MS (MALDI-MS): [M]+ calcd for $C_{74}H_{90}N_4O_2S$ 1098.7, found 1098.5.

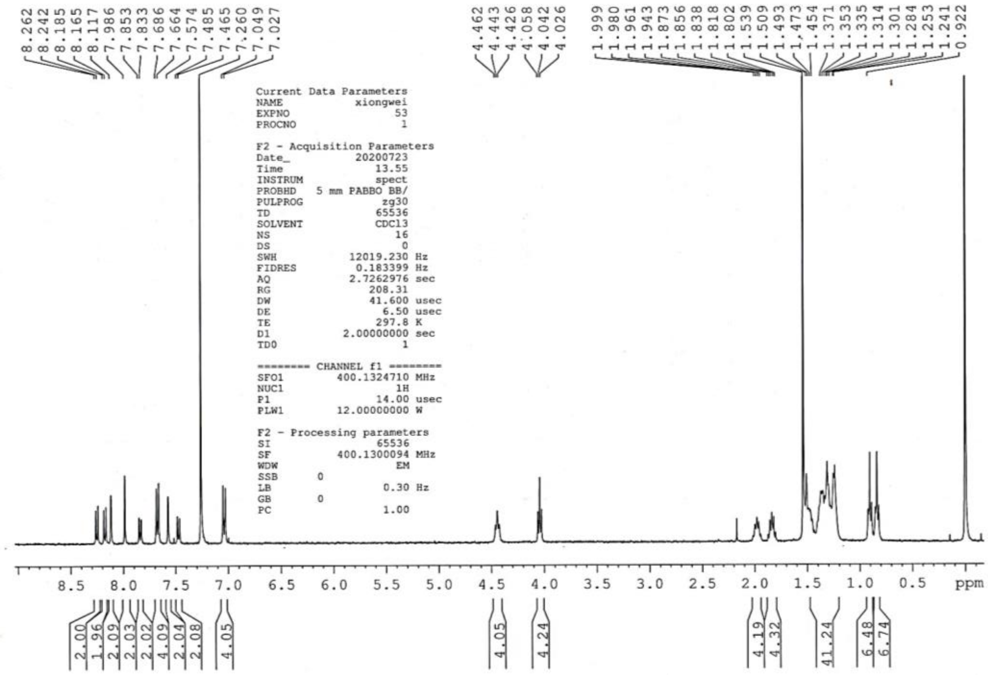

Supplementary Figure 1. ^{1}H NMR spectrum of BPCBT.

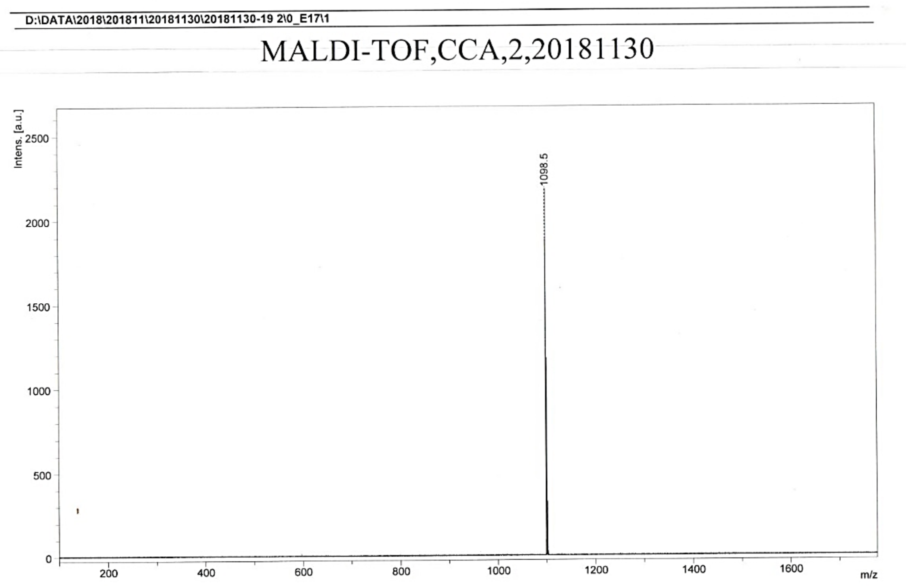

Supplementary Figure 2. MALDI-MS of BPCBT.

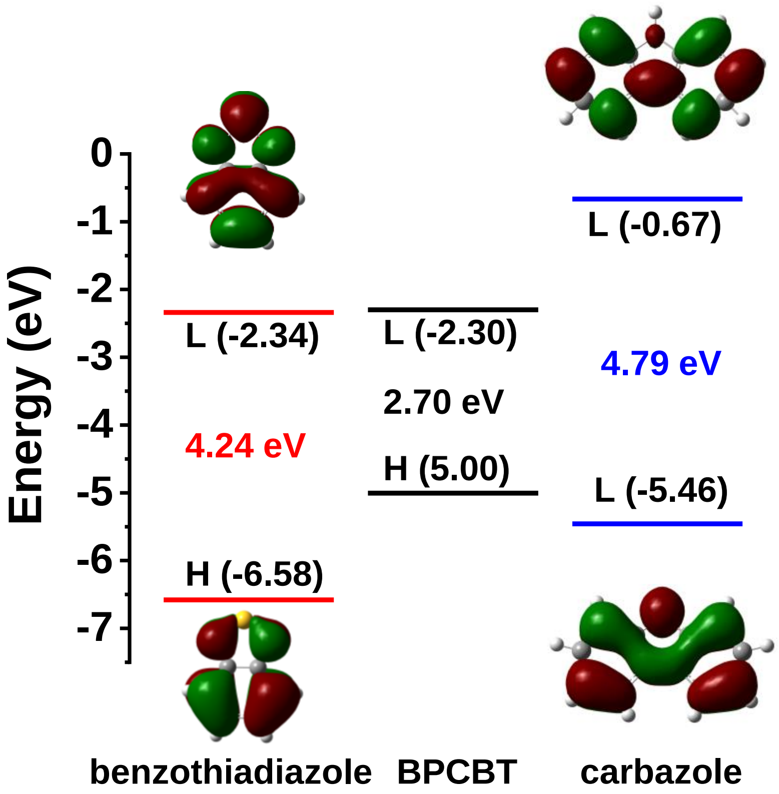

Supplementary Figure 3. Molecular orbitals and energy level diagrams of benzothiadiazole, BPCBT and carbazole.

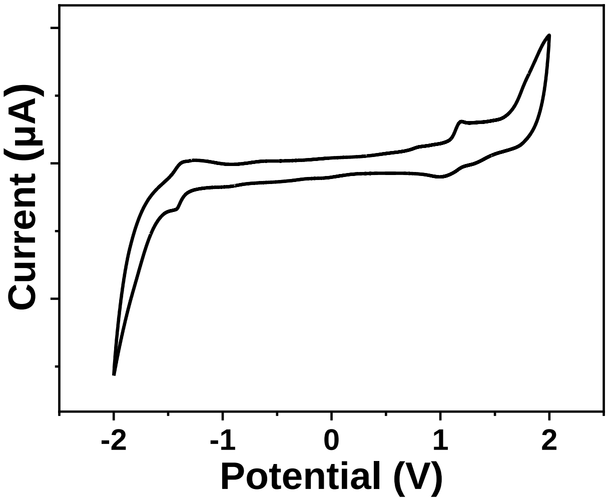

Supplementary Figure 4. Cyclic voltammograms of BPCBT (0.05 M) in CH_2Cl_2 solution, with Pt disk and Pt wire as the working and counter electrodes, Ag/AgCl electrode (sat. KCl) as the reference electrode, and *n*-Bu4NPF6 (0.1 M) as supporting electrolyte.

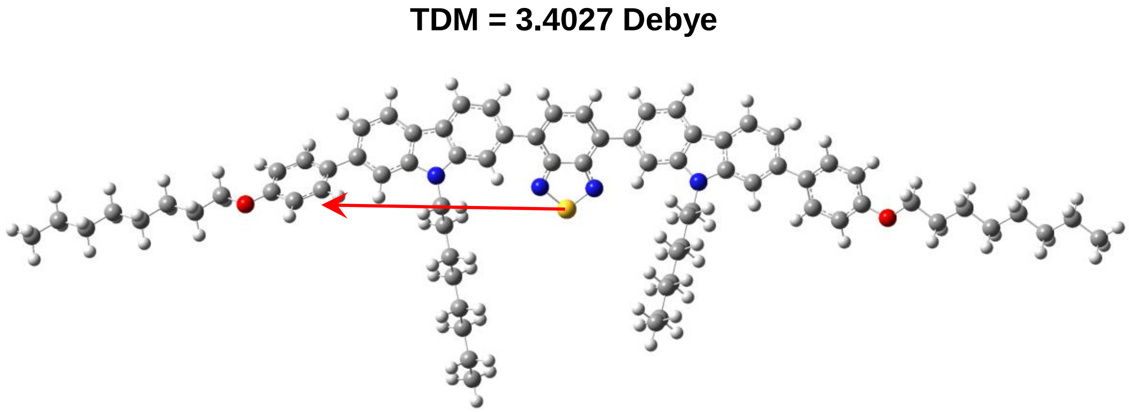

Supplementary Figure 5. Transition dipole moment (TDM) (μ_x = -3.3996 D, μ_y = -0.0023 D, μ_z = -0.1452 D) of BPCBT. The direction of TDM is nearly parallel to the long axis of the molecule.

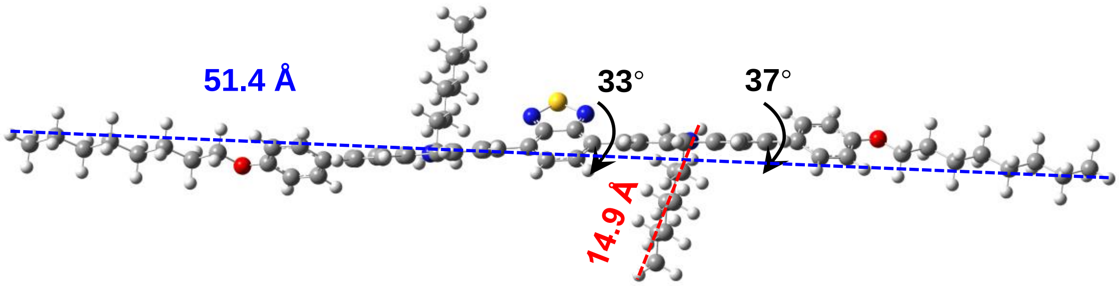

Supplementary Figure 6. Dihedral angles and molecular lengths calculated from the optimized structure of BPCBT.

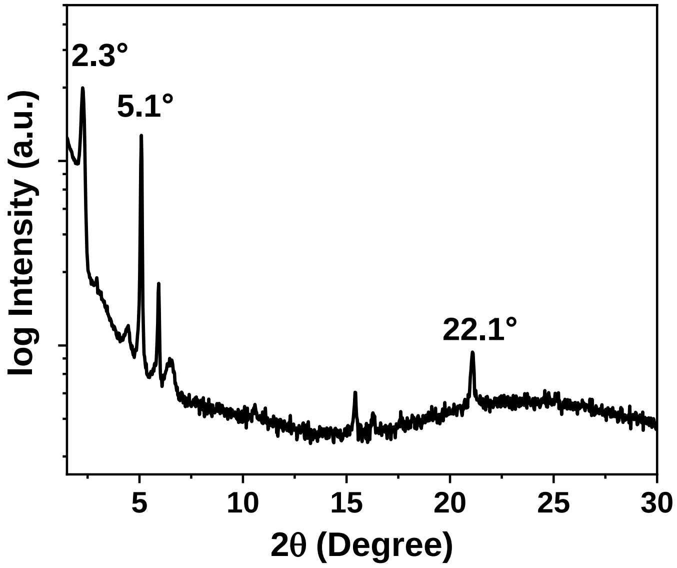

Supplementary Figure 7. XRD pattern of BPCBT microwires deposited on glass substrate.

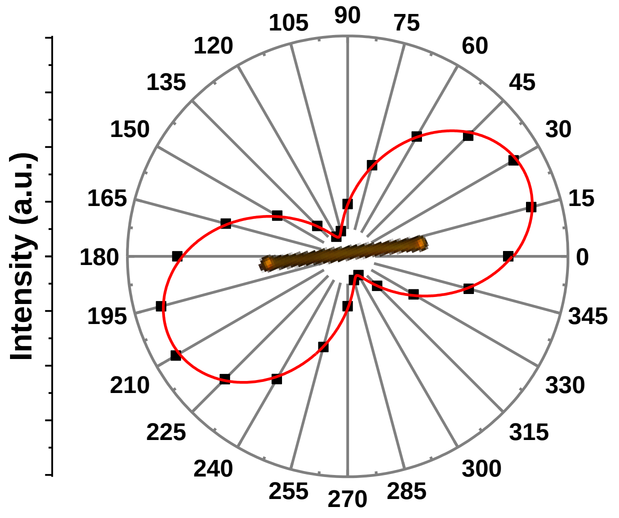

Supplementary Figure 8. Fluorescence intensity versus the polarization angle of excitation light measured from a single BPCBT microwire.

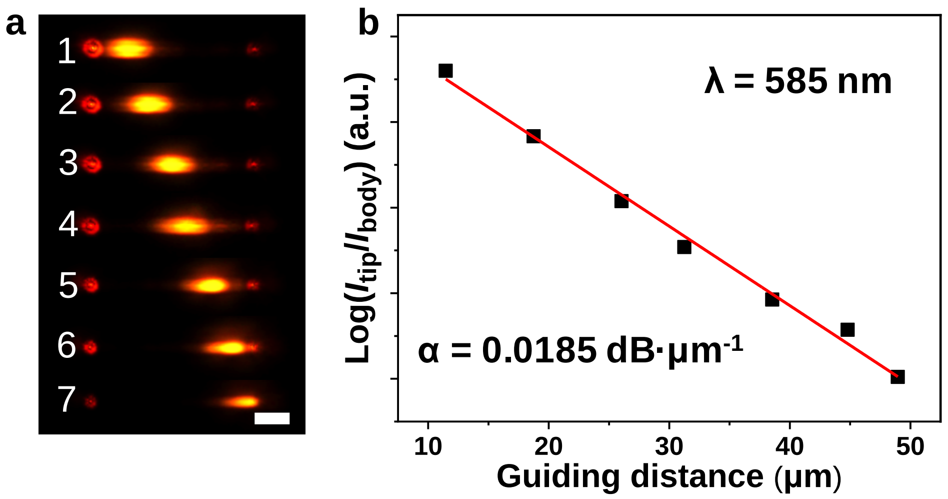

Supplementary Figure 9. a Fluorescence images obtained from the BPCBT microwire excited at different positions. Scale bar is 10 μm. **b** The plot of log(I_{tip}/I_{body}) against the guiding distance. The calculated optical loss coefficient (α) at 585 nm is 0.0185 dB·μm^{-1}.

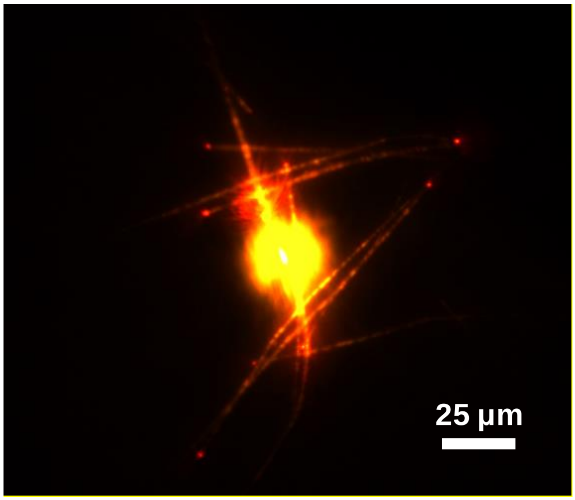

Supplementary Figure 10. Fluorescence image of coupled BPCBT microwires under local excitation. Light signals could be efficiently coupled to neighboring microwires.

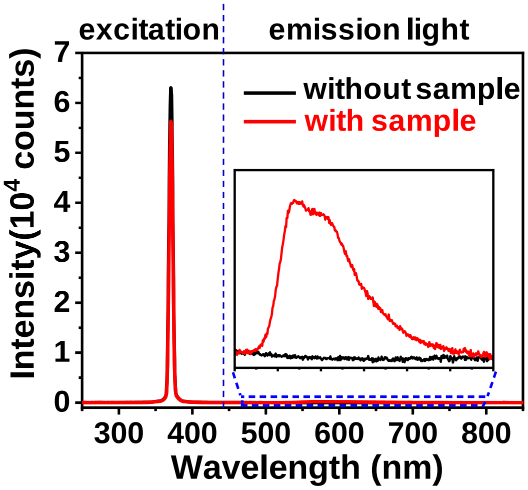

$$\boldsymbol{\Phi} = \frac{\mathbf{A}_{\mathbf{em}}}{\mathbf{A}_{\mathbf{ex,\ with\ sample}} - \mathbf{A}_{\mathbf{ex,without\ sample}}}$$

Sample	Quantum yields (Φ)
solution	0.98
microwire	0.81

Supplementary Figure 11. Absolute fluorescence quantum yields (Φ) of BPCBT monomers and microwires. A_{em} is the integrated area of the emission spectra of the sample. $A_{ex,\ with\ sample}$ and $A_{ex,\ without\ sample}$ are the integrated area of the excitation light at 370 nm with and without sample, respectively.

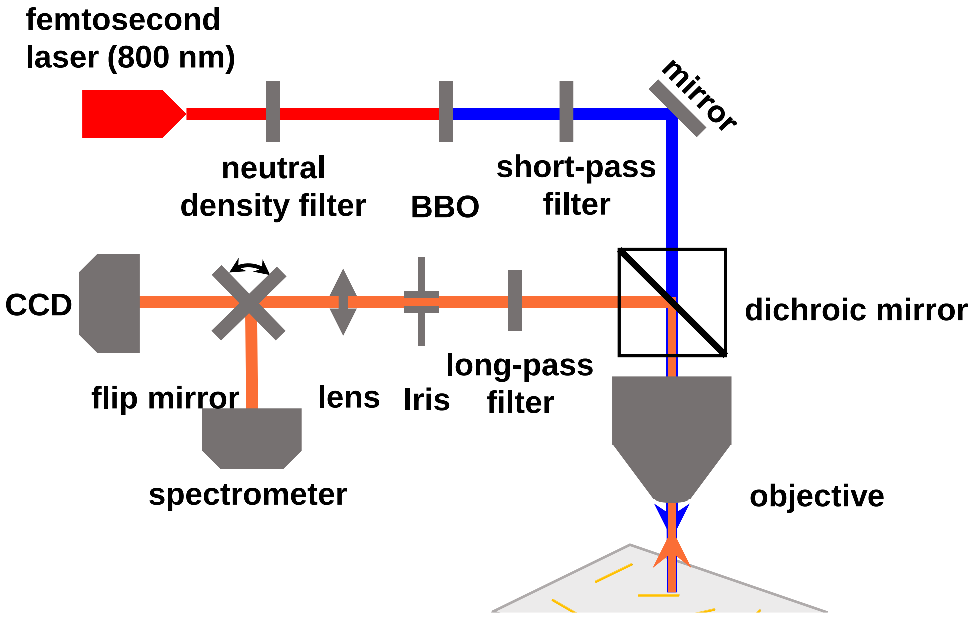

Supplementary Figure 12. Schematic illustration of the home-built setup for optical characterization.

The excitation laser pulses (400 nm, ~200 fs, 1 kHz) were generated from the second harmonic of the fundamental output of a regenerative amplifier (Solstice, Spectra-Physics, 800 nm, ~200 fs, 1 kHz). The pulsed laser was then conducted into an ×20 objective lens focusing down to a 25 μm diameter spot to locally excite the BPCBT microwire, and the light emission was collected with the objective and filtered with a 420 nm long pass filter. The power at the input was altered by a neutral density filter, and the pump energy was measured on the objective lens. The emission spectrum was measured with a grating spectrometer (Acton SP-2358) equipped with a thermal-electrically cooled CCD (Princeton Instruments ProEM:1600).

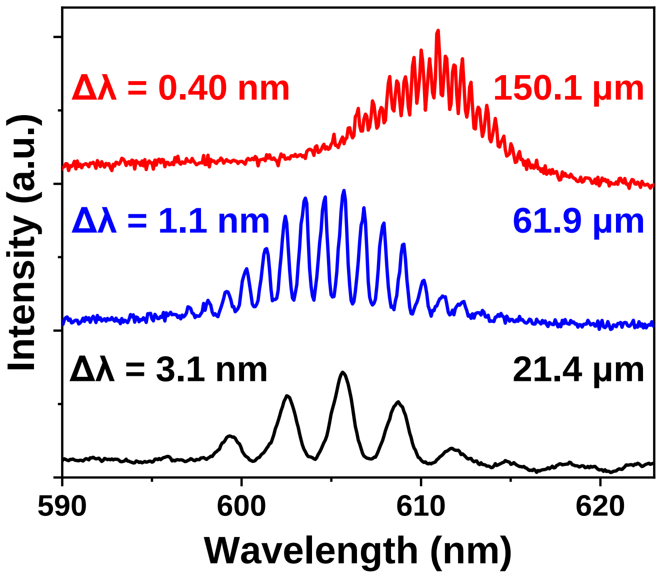

Supplementary Figure 13. Lasing spectra above threshold from BPCBT microwires with various lengths.

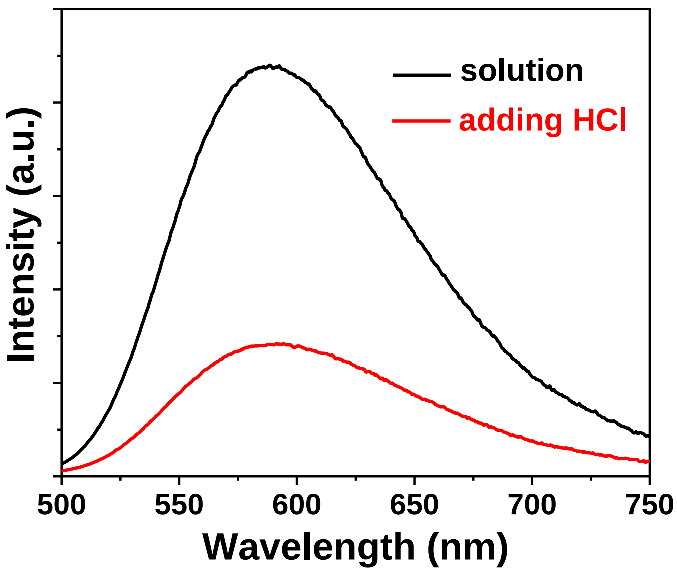

Supplementary Figure 14. Photoluminescence spectra from BPCBT solution before and after adding HCl.

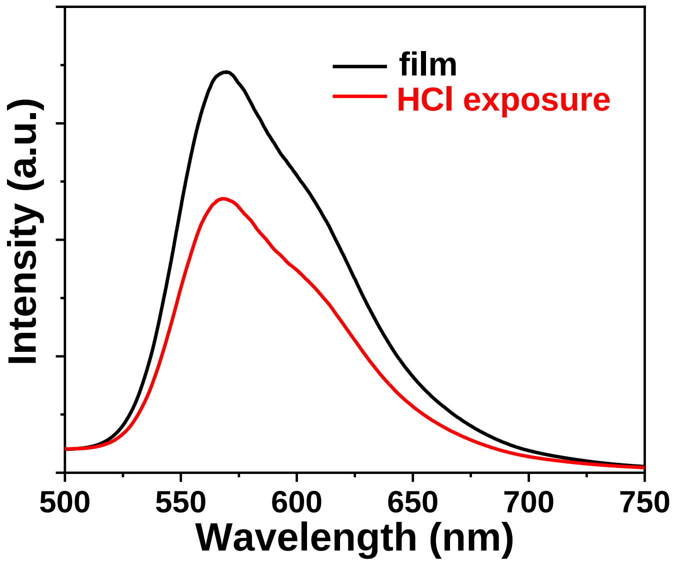

Supplementary Figure 15. Photoluminescence spectra from BPCBT thin film before and after exposure to HCl vapor.

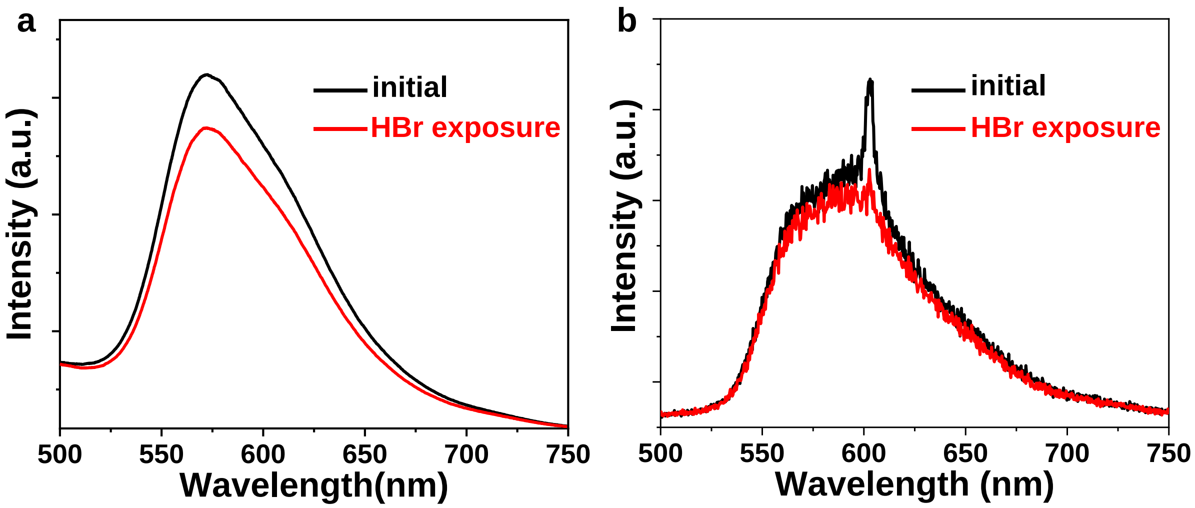

Supplementary Figure 16. a Photoluminescence and **b** lasing spectra from BPCBT microwires before and after exposure to HBr vapor.

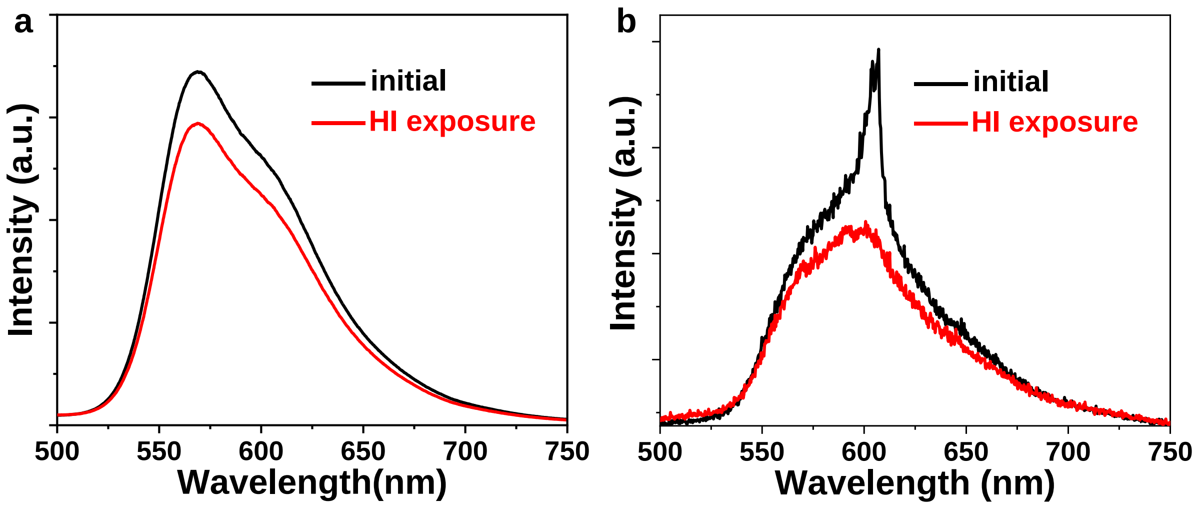

Supplementary Figure 17. a Photoluminescence and **b** lasing spectra from BPCBT microwires before and after exposure to HI vapor.

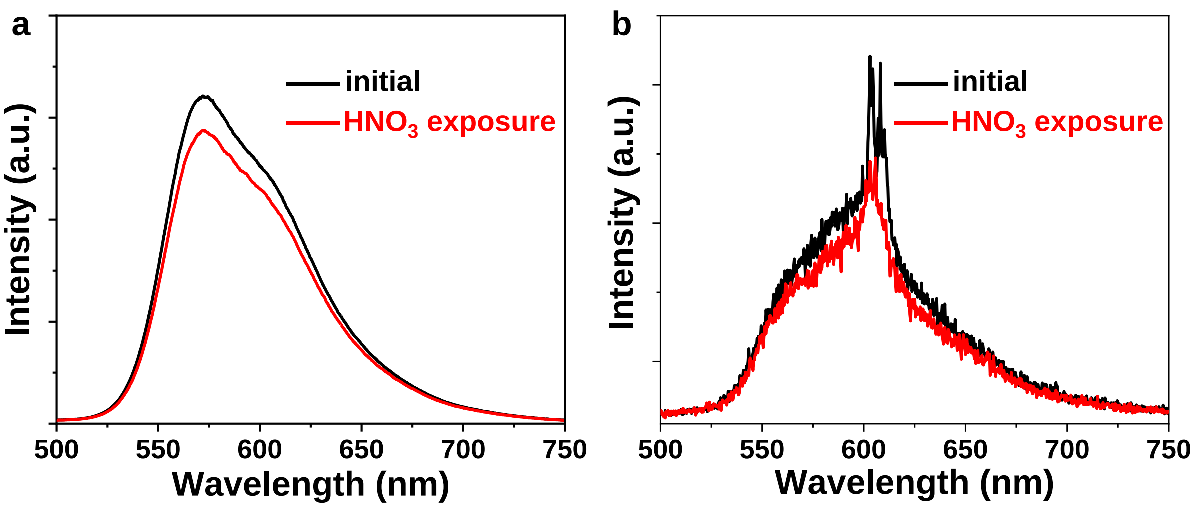

Supplementary Figure 18. a Photoluminescence and **b** lasing spectra from BPCBT microwires before and after exposure to HNO_3 vapor.

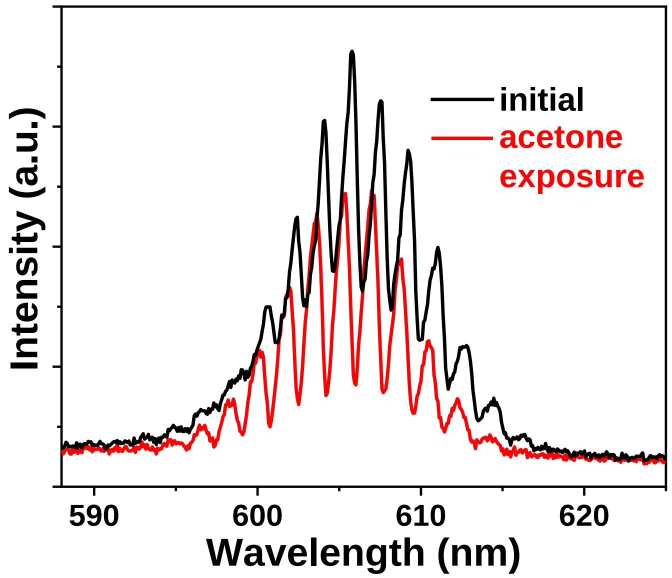

Supplementary Figure 19. Lasing spectra of a BPCBT microwire before and after exposure to acetone vapor.

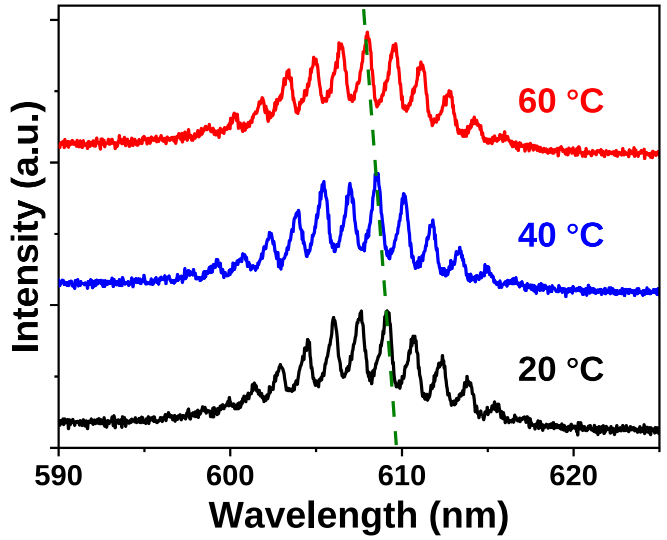

Supplementary Figure 20. Lasing spectra from a BPCBT microwire at different temperatures.

Supplementary Table 1. Laser actions in carbozole derivatives and BPCBT.

Compound	Emission wavelength (nm)	Pump source	Condition	Threshold
MC6Cz-2-NPC[a]	435	-, 4.2 ns, 10 Hz	film	0.145 $mJ\cdot cm^{-2}$
9,9'-{2,1,3-benzothiadiazole-4,7-diylbis[(ethyne-2,1-diyl)-4,1-phenylene]}bis(9H-carbazole)[b]	509	436 nm, 10 ns, 10Hz	doped in PS	40 $kW\cdot cm^{-2}$ (4 $mJ\cdot cm^{-2}$)
HBT-Cz[c]	512	337 nm, 3.5 ns, 20 Hz	solution	21 $mJ\cdot cm^{-2}$
G1COPV2[d]	488	397 nm, 300 fs, 1 kHz	microrod	0.32 $mJ\cdot cm^{-2}$
G3COPV2[d]	496	397 nm, 300 fs, 1 kHz	grain	0.3 $mJ\cdot cm^{-2}$
2-(9H-carbazol-2-yl)benzo[d]thiazole[e]	433	355 nm, 10 ns, 10Hz	solution	24.2 $mJ\cdot cm^{-2}$
2-(9-ethyl-9H-carbazol-2-yl)benzo[d]thiazole[e]	435	355 nm, 10 ns, 10Hz	solution	6.1 $mJ\cdot cm^{-2}$
BPCBT (this work)	606	400 nm, 200 fs, 1 kHz	microwire	**1.6 $mJ\cdot cm^{-2}$**

a. Han, Y. M. et al. Solution-processed diarylfluorene derivatives for violet-blue amplified spontaneous emission and electroluminescence. *J. Mater. Chem. C* **5**, 9903-9910 (2017).

b. Martín, R. et al. Design, synthesis and amplified spontaneous emission of 1,2,5-benzothiadiazole derivatives. *J. Mater. Chem. C* **7**, 9996-10007 (2019).

c. Mai, V. N. et al. Low amplified spontaneous emission threshold and efficient electroluminescence from a carbazole derivatized excited-state intramolecular proton transfer dye. *ACS Photonics* **5**, 4447-4455 (2018).

d. Iwai, K. et al. Single-crystalline optical microcavities from luminescent dendrimers. *Angew. Chem.* **132**, 12774-12779 (2020).

e. Yang, Z. X., Tian, Y. Q., Cang, S., Zhang, L. Y. & Liu, L. The synthesis of a series of fluorescent emitters and their application for dye lasing and cation sensing. *Spectrochim. Acta, Part A* **246**, 118978 (2021).
