# Supplementary material for: Stable organic self-assembled microwire lasers for chemical vapor sensing
Source: Commun Chem. 2021 Jun 24;4:97. doi: 10.1038/s42004-021-00534-x (PMC9814925; doi:10.1038/s42004-021-00534-x)
Supplement: Supplementary file 2 — Reporting Summary [file 42004_2021_534_MOESM2_ESM.pdf]

nature research

Corresponding author(s): Chuang Zhang

Lasing Reporting Summary

Nature Research wishes to improve the reproducibility of the work that we publish. This form is intended for publication with all accepted papers reporting claims of lasing and provides structure for consistency and transparency in reporting. Some list items might not apply to an individual manuscript, but all fields must be completed for clarity.

For further information on Nature Research policies, including our data availability policy, see Authors & Referees.

Experimental design

Please check: are the following details reported in the manuscript?

1. Threshold

Item	Yes	No	Details
Plots of device output power versus pump power over a wide range of values indicating a clear threshold	☒	☐	Figure. 3b

2. Linewidth narrowing

Item	Yes	No	Details
Plots of spectral power density for the emission at pump powers below, around, and above the lasing threshold, indicating a clear linewidth narrowing at threshold	☒	☐	Figure. 3a and Figure. 3b.
Resolution of the spectrometer used to make spectral measurements	☒	☐	Page 12 in Supplementary Information.

3. Coherent emission

Item	Yes	No	Details
Measurements of the coherence and/or polarization of the emission	☐	☒	This manuscript focuses on the enhanced sensing based on lasing intensity. The coherent and polarization measurements have less correlations with the main content.

4. Beam spatial profile

Item	Yes	No	Details
Image and/or measurement of the spatial shape and profile of the emission, showing a well-defined beam above threshold	☒	☐	Figure. 3a and Figure. 4b.

5. Operating conditions

Item	Yes	No	Details
Description of the laser and pumping conditions<br>*Continuous-wave, pulsed, temperature of operation*	☒	☐	Page 12 in Supplementary Information.
Threshold values provided as density values (e.g. W cm^{-2} or J cm^{-2}) taking into account the area of the device	☒	☐	Paragraph 2, Page 4 and Page 12 in Supplementary Information.

6. Alternative explanations

Item	Yes	No	Details
Reasoning as to why alternative explanations have been ruled out as responsible for the emission characteristics<br>*e.g. amplified spontaneous, directional scattering; modification of fluorescence spectrum by the cavity*	☒	☐	Paragraph 2, Page 4.

7. Theoretical analysis

Item	Yes	No	Details
Theoretical analysis that ensures that the experimental values measured are realistic and reasonable<br>*e.g. laser threshold, linewidth, cavity gain-loss, efficiency*	☒	☐	Paragraph 2, Page 4.

8. Statistics

Item	Yes	No	Details
Number of devices fabricated and tested	☒	☐	Paragraph 2, Page 5.
Statistical analysis of the device performance and lifetime (time to failure)	☒	☐	Paragraph 3, Page 4.
